# Receptor Activator of NF-kB (RANK) Expression in Primary Tumors Associates with Bone Metastasis Occurrence in Breast Cancer Patients

**DOI:** 10.1371/journal.pone.0019234

**Published:** 2011-04-29

**Authors:** Daniele Santini, Gaia Schiavon, Bruno Vincenzi, Laura Gaeta, Francesco Pantano, Antonio Russo, Cinzia Ortega, Camillo Porta, Sara Galluzzo, Grazia Armento, Nicla La Verde, Cinzia Caroti, Isabelle Treilleux, Alessandro Ruggiero, Giuseppe Perrone, Raffaele Addeo, Philippe Clezardin, Andrea Onetti Muda, Giuseppe Tonini

**Affiliations:** 1 Department of Medical Oncology, University Campus Bio-Medico of Rome, Rome, Italy; 2 Department of Medical Oncology, Erasmus University Medical Center - Daniel den Hoed Cancer Center, Rotterdam, The Netherlands; 3 Department of Pathology, University Campus Bio-Medico of Rome, Rome, Italy; 4 Section of Medical Oncology, Department of Surgical and Oncological Sciences, Palermo University, Palermo, Italy; 5 Division of Medical Oncology and Haematology, Institute for Cancer Research and Treatment (IRCC), Candiolo, Italy; 6 Department of Medical Oncology, IRCCS San Matteo University Hospital Foundation, Pavia, Italy; 7 Department of Oncology, Azienda Ospedaliera-Ospedale Fatebenefratelli e Oftalmico, Milan, Italy; 8 S.C.Medical Oncology, Ente Ospedaliero Ospedali Galliera, Genova, Italy; 9 INSERM, Research Unit U664, University of Lyon-1, Lyon, France; 10 Department of Radiology, Erasmus Medical Center, Rotterdam, The Netherlands; 11 Department of Medical Oncology, “San Giovanni di Dio” Hospital, Frattaminore, Naples, Italy; Virginia Commonwealth University, United States of America

## Abstract

**Background:**

Receptor activator of NFkB (RANK), its ligand (RANKL) and the decoy receptor of RANKL (osteoprotegerin, OPG) play a pivotal role in bone remodeling by regulating osteoclasts formation and activity. RANKL stimulates migration of RANK-expressing tumor cells in vitro, conversely inhibited by OPG.

**Materials and Methods:**

We examined mRNA expression levels of RANKL/RANK/OPG in a publicly available microarray dataset of 295 primary breast cancer patients. We next analyzed RANK expression by immunohistochemistry in an independent series of 93 primary breast cancer specimens and investigated a possible association with clinicopathological parameters, bone recurrence and survival.

**Results:**

Microarray analysis showed that lower RANK and high OPG mRNA levels correlate with longer overall survival (P = 0.0078 and 0.0335, respectively) and disease-free survival (P = 0.059 and 0.0402, respectively). Immunohistochemical analysis of RANK showed a positive correlation with the development of bone metastases (P = 0.023) and a shorter skeletal disease-free survival (SDFS, P = 0.037). Specifically, univariate analysis of survival showed that “RANK-negative” and “RANK-positive” patients had a SDFS of 105.7 months (95% CI: 73.9–124.4) and 58.9 months (95% CI: 34.7–68.5), respectively. RANK protein expression was also associated with accelerated bone metastasis formation in a multivariate analysis (P = 0.029).

**Conclusions:**

This is the first demonstration of the role of RANK expression in primary tumors as a predictive marker of bone metastasis occurrence and SDFS in a large population of breast cancer patients.

## Introduction

Bone is the most common site of metastatic invasion in breast cancer. Skeletal metastases from breast cancer are mostly osteolytic, with histological evidence of increased number and activity of bone-resorbing osteoclasts. However, the molecular mechanisms of breast cancer metastasis to the skeleton are still poorly understood. Recently, a novel cytokine triad consisting of receptor activator of NF-kB ligand (RANKL), its receptor (RANK) and the endogenous decoy receptor osteoprotegerin (OPG) was identified and extensively characterized for its role in bone remodeling. It is well known that RANK/RANKL/OPG axis controls osteoclastogenesis and bone resorption [1].

The TNF ligand superfamily member RANKL, which is expressed on the surface of osteoblasts, is critical for the formation, function and survival of osteoclasts [2], [3]. It exerts its functions by binding and activating its receptor RANK [4], [5], which is expressed on the surface of osteoclastic precursors and mature osteoclasts [6]. OPG is a soluble member of the TNF receptor super family secreted by osteoblasts which, by competing with RANK for binding to RANKL, acts as a decoy receptor, thereby inhibiting osteoclastogenesis [7]. Alterations of the RANKL/OPG balance have been reported in a spectrum of skeletal diseases characterized by excessive osteoclastic activity, including osteoporosis, rheumatoid arthritis and bone metastases. RANK expression is not restricted to bone, as it is also observed in other tissues including breast, lung, brain, kidney and cartilage. Moreover, the RANKL/RANK/OPG system is disregulated in several tumors, such as breast cancer, malignant bone tumors, multiple myeloma, giant cell tumors of bone, chondroblastoma, neuroblastoma and squamous cell carcinoma [8]–[14]. Recently, functional RANK expression was reported in cancer cell lines from human origin (osteosarcoma, breast and prostate carcinomas) [15], [16], and in mouse melanoma cell lines [16]. RANK/RANKL expression was also found in resected specimens obtained from breast, hepatocellular and prostate cancer and multiple myeloma. In breast, RANKL and RANK are expressed in the normal tissue and, conversely to RANK, an apparent loss of RANKL expression occurs in neoplastic tissue. However, breast tumors retaining RANKL expression tend to be less differentiated and estrogen receptor negative [17]. In prostate, RANKL/RANK expression is low in normal tissue but high in neoplastic tissues and even higher in metastatic lesions [18], [19]. Finally, Sasaki et al examined cases of primary hepatocellular carcinoma (HCC), showing that RANKL expression in HCC cells correlated with the development of bone metastasis after hepatic resection [20].

On the basis of a high constitutive RANK expression in breast cancer specimens and cell lines, recent data suggest that the RANK status in cancer cells determines their tendency to metastasize to bone whereas RANKL is abundantly expressed [16]. This hypothesis is supported by the observation that RANKL induces the migration of various RANK-expressing cancer cell lines in vitro, in a manner that is blocked by RANKL inhibitors (OPG or sRANK/RANK-Fc). Moreover, OPG treatment of tumor-bearing animals prevents the homing of RANK-expressing B16F10 melanoma cells in bone [16]. Moreover, increased RANKL expression is related to migration and metastatic propensity of prostate tumor cells and renal cell carcinomas [21], [22]. Thus, the RANKL/RANK pathway may dictate breast cancer cells to preferentially migrate into bone. To the best of our knowledge, there are not previous studies which investigated a large and homogeneous cohort of breast cancer patients about the role of RANK expression in primary cancer cells in predicting bone metastatization.

Specifically, the main purpose of our research was to demonstrate the potential role of RANK expression in primary tumors as a predictive marker of bone metastasis occurrence and skeletal disease-free survival (SDFS) in a population of breast cancer patients.

## Results

### Microarray analysis: RANK/RANKL/OPG pathway and clinicopathological prognostic factors

**Figure 1:**
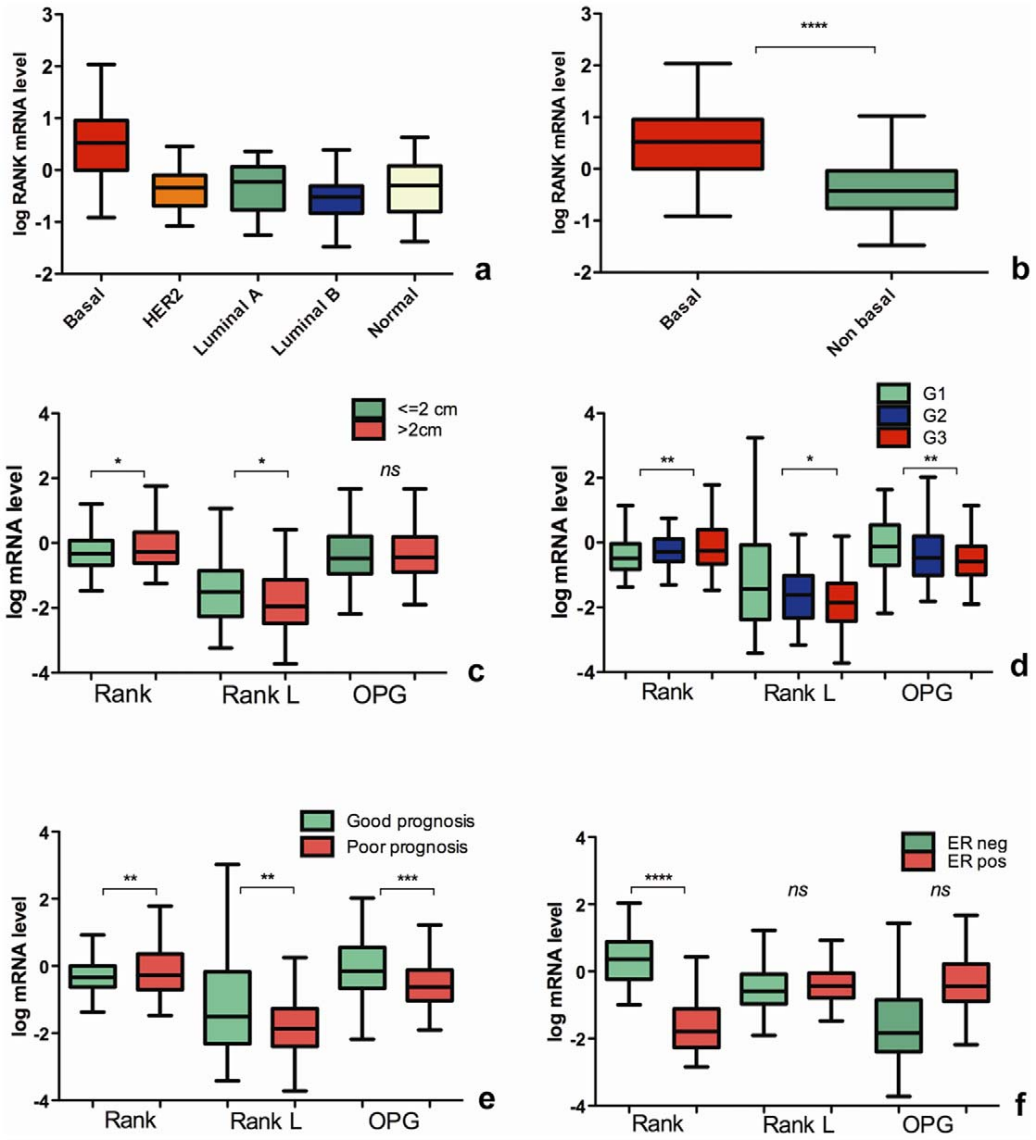
RANK/RANKL/OPG pathway, molecular and clinicopathological prognostic factors in 295 early breast cancer patients from NKI microarray dataset. Tukey whisker plots indicate the correlation between normalized mRNA level of RANK/RANKL/OPG and prognostic factors in a cohort of 295 primary breast cancer patients from NKI (34). **Panel A**: basal tumors present a higher expression of RANK mRNA compared to all the other molecular subtypes. **Panel B**: this difference is strongly significant comparing basal- versus non basal-type tumors. **Panel C**: we found a statistically significant difference between tumor ≤2 cm and >2 cm in RANK and RANKL. **Panel D**: RANK/RANKL/OPG are strongly associated with histological grading. **Panel E**: dividing the population in “poor prognosis group” and “good prognosis group” on the basis of the 70-gene signature, RANK expression is significantly higher in the “poor prognosis” versus “good prognosis” group, while RANKL and OPG levels are higher in the “good prognosis” versus “poor prognosis”. **Panel F**: RANK expression is significantly higher in ER negative tumors. RANKL and OPG do not show a significant difference between the two groups. *P* values are indicated as: * *P* = <0.05, ** *P* = <0.01, *** *P*<0.001, **** *P*<0.0001.

The correlation between RANK, OPG and RANKL levels with the clinico-molecular prognostic factors available for the NKI cohort (Table 1) was analyzed. On the basis of RANK level distribution, basal tumors have higher expression compared to all the other molecular subtypes (Figure 1A) and the difference is strongly significant (*P*<0.0001) comparing basal- versus non basal-type tumors (Figure 1B).

**Table 1:** Clinico-molecular characteristics of 295 breast cancer patients from NKI microarray dataset.

Parameter	pts (n)	pts (%)
***Age (ys)***		
<50	246	83.4
≥50	49	16.6
***Positive nodes (n)***		
0	151	51.2
1–3	106	35.9
≥4	38	12.9
***Size (cm)***		
≤2	155	52.5
>2	140	47.5
***Histological grade***		
Well differentiated (G1)	75	25.5
Intermediate (G2)	101	34.2
Poor (G3)	119	40.3
***ER status***		
Negative	69	23.4
Positive	226	76.6
***70-gene signature***		
Poor prognosis	180	61
Good prognosis	115	39

Clinico-molecular characteristics of 295 breast cancer patients from NKI cohort (24). Raw data and complete clinical data were downloaded from the Rosetta Web site (http://www.rii.com).

No correlation was found between age (<50 and ≥50 years) and the expression of any of the three molecules (data not shown). Regarding tumor size, we found a statistically significant difference between tumor ≤2 cm and >2 cm in RANK and RANKL at transcriptional level (Figure 1C), whereas OPG levels are not significantly correlated with size. All the three parameters are strongly associated with histological grading (Figure 1D). RANK levels are lower in well differentiated (G1) than intermediate (G2) and poorly differentiated (G3) tumors. In particular, a statistically significant difference is observed between G1 and G3 (*P*<0.01). Conversely, OPG and RANKL level are higher in G1 tumors than G2 and G3 (*P*<0.05 for RANKL and *P*<0.01 for OPG, comparing G1 and G3). Interestingly, dividing the population in “poor prognosis” and “good prognosis” group on the basis of the 70-gene signature, RANK expression is significantly higher in the “poor” versus “good prognosis” group (*P* = 0.01), while RANKL and OPG levels are higher in the “good prognosis” versus “poor prognosis” (*P* = 0.003 and 0.0005, respectively) (Figure 1E). Importantly, ER negative tumors RANK expression is significantly higher (*P*<0.0001), but RANKL and OPG do not show a significant difference between the two groups (Figure 1F).

### Univariate analysis of survival in microarray study

**Figure 2:**
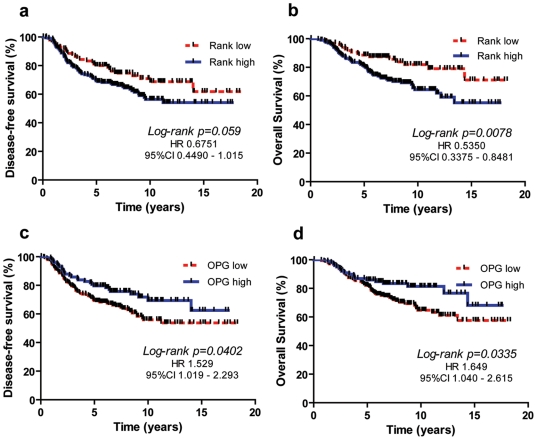
Univariate analysis of survival in microarray study. 295 primary breast cancers from NKI microarray dataset (34) were divided in three equally numerous groups depending on the level of mRNA expression of RANK and OPG (high, medium or low). Tumors with high and medium RANK level were grouped together and are indicated as possessing high RANK mRNA (H) vs low (L). OPG expression was dichotomized as low (L+M) vs high (H). The Kaplan-Meier curves show the correlation between expression of RANK or OPG and DFS and OS. The curves were analyzed by Log-rank (Mantel-Cox) Test, Hazard ratios and 95% confidence intervals (CIs) were calculated by use of a stratified Cox regression analysis. **Panel A and B**: patients with low level of RANK show a better DFS (*P* = 0.059) and a statistically significant longer OS (*P* = 0.0078), compared to patients with high RANK expression. **Panel C and D**: patients with higher mRNA level of OPG present a longer DFS (*P* = 0.0402) and OS (*P* = 0.0335) than patients with lower OPG.

Kaplan Meier curve showed a significant correlation between low RANK levels and longer overall survival (OS) (*P* = 0.0078) (Figure 2B). Patients with low level of RANK tend to have a better disease free survival (DFS), although the significance is not observed in this dataset (*P* = 0.059) (Figure 2A). Regarding RANKL expression, we did not find any statistically significant correlation neither with DFS nor OS (data not shown). Patients with higher level of OPG present a longer DFS (*P* = 0.040) and OS (*P* = 0.033) than patients with lower OPG (Figure 2C and 2D).

### Experimental phase: RANK expression and clinicopathological characteristics in breast cancer patients

**Figure 3:**
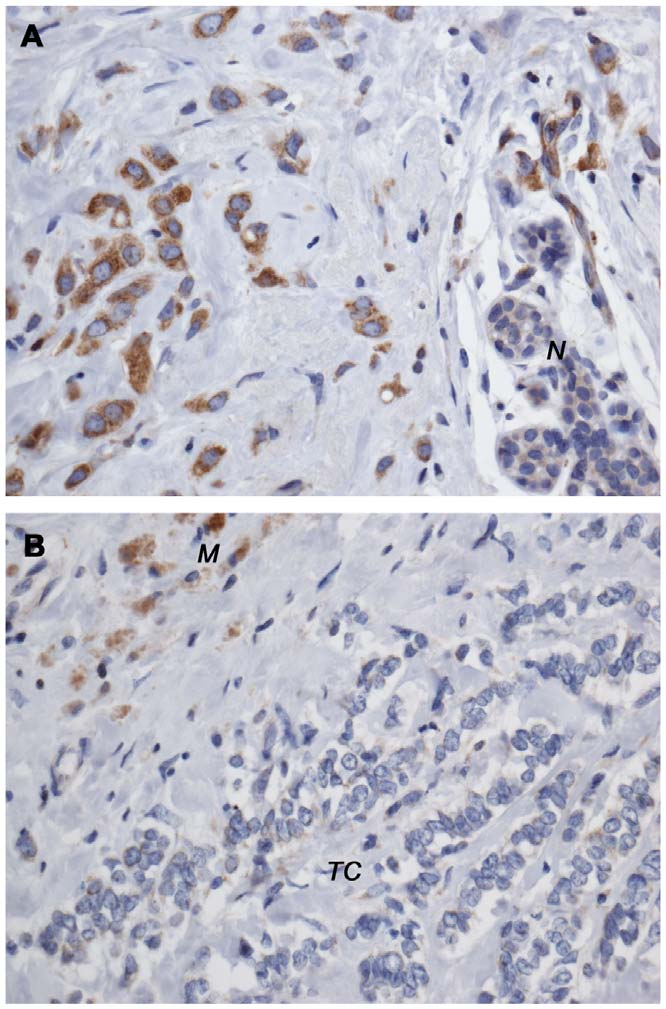
Immunohistochemical results in 93 breast cancers. **Image A**: RANK positive breast cancer. The normal glandular epithelium is negative (*N*) confirming the RANK protein overexpression of the tumor tissue. **Image B**: RANK negative breast cancer (*TC*). Tissue-associated macrophages (*M*) are positive for RANK staining and are used as internal positive controls. Original magnification A,B 200×.

Among RANKL/RANK/OPG, RANK resulted to be more strikingly and consistently correlated both with clinical prognostic factors and survival. Therefore, we performed IHC for RANK in our independent series of 77 IDC samples and 15 ILC samples. RANK overexpression was found in 39% (30/77) IDC and in 53% (8/15) ILC samples (Figure 3, Table 2). 35% (22/63) patients with N0 or N1 node status were RANK-positive, whereas 55% (16/29) patients with N2 or N3 node status were RANK-positive. The only G1 sample was RANK-negative, whereas 35% (15/43) of G2 samples and 55% (16/43) of G3 samples were positive for RANK expression. Only 36% (9/25) HER2/neu positive samples were RANK-positive, whereas 40% (22/55) HER2/neu negative samples were RANK-positive and 54% (7/13) patients without a known HER2/neu were RANK-positive. 31% (10/32) samples from cases without metastases were RANK-positive, whereas 31% (5/16) samples from patients with visceral metastases were positive for expression of RANK in the tumor cells. Moreover, 31% (5/16) samples from patients with bone metastasis were RANK-positive as well as 62% (18/29) samples from patients with bone and visceral involvement. RANK expression was independent from histotype (*P* = 0.23), HER2/neu expression (*P* = 0.47) and grading (*P* = 0.39), but his positivity was associated with lymph nodes involvement (*P* = 0.05). However, RANK-positive patients showed a significantly higher risk to develop skeletal metastases (*P* = 0.023) (Table 2).

Moreover, RANK positivity was detected in 51.1% (23/43) of patients who developed metastases versus 31.25% (15/48) of patients who remained metastasis-free (P = 0.042).

**Table 2:** Correlation between clinico-pathologic parameters and RANK expression in 93 patients with IDC or ILC.

Parameter	pts (n)	RANK+(%)	*P*
***Histological type***			
Ductal	77	30 (39)	0.23
Lobular	15	8 (53)	
Unknown	1	0 (0)	
***Positive nodes (n)***			
N0–N1	63	22 (35)	**0.05**
N2–N3	29	16 (55)	
Unknown	1	0 (0)	
***Histological grade***			
Well differentiated (G1)	1	0 (0)	0.39
Intermediate (G2)	43	15 (35)	
Poor (G3)	43	16 (37)	
Unknown	6	0 (0)	
***Hercep test***			
Positive	25	9 (36)	0.47
Negative	55	22 (40)	
Unknown	13	7 (54)	
***Sites of Metastasis***			
Bone	16	5 (31)	**0.023**
Visceral	16	5 (31)	
Bone+Visceral	29	18 (62)	
No metastasis	32	10 (31)	

Clinico-molecular characteristics of patients included in our independent series of 93 primary breast cancer specimens used to perform RANK IHC. RANK positivity is associated with lymph nodes involvement (*P* = 0.05). RANK-positive patients show a significantly higher risk to develop skeletal metastases (*P* = 0.023). The association between variables was performed using the chi square test. IDC: intraductal carcinoma; ILC: intralobular carcinoma.

IDC: intraductal carcinoma; ILC: intralobular carcinoma; pts: patients; ys: years.

### Correlation of clinicopathological data with SDFS (univariate analysis)

Univariate survival analysis did not show correlation of histotype, grading, HER2/neu status with SDFS (data not shown). Only nodes involvement was significantly associated with an early skeletal recurrence (*P* = 0.012).

### RANK expression associates with accelerated bone metastasis (univariate analysis)

**Figure 4:**
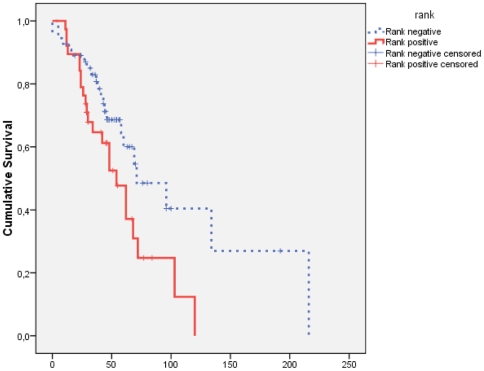
RANK expression associates with accelerated bone metastasis in 93 breast cancer patients (Kaplan Meyer curves of SDFS). RANK negative patients showed a SDFS of 105.7 months (95% C.I: 73.9–124.4) compared with only 58.9 months (95% C.I: 34.7–68.5) in RANK positive patients. The difference is statistically significant (*P* = 0.034).

To establish the skeletal prognostic role of RANK, patients were stratified in positive (RANK-high expression) versus negative (RANK-low expression). Univariate analysis of survival showed that the RANK expression was associated with SDFS. Specifically, patients with overexpression of RANK showed a shorter SDFS 58.9 months (95% C.I: 34.7–68.5) than RANK negative patients 105.7 months (95% C.I: 73.9–124.4) (*P* = 0.034; Figure 4).

### Multivariate analysis

Multivariate analysis using the Cox proportional hazards model, demonstrated that both nodes status and RANK expression are independent prognostic indicators for early bone metastasis development (*P* = 0.029 and 0.037, respectively) (Table 3).

**Table 3:** Multivariate analysis of skeletal disease free survival.

	RR	95% C.I.	*P*
**RANK**			
High Expression	1	-	**0.037**
Low expression	0.211	0.120–0.855	
***Nodal Status***			
N1	1	-	**0.029**
N0	0.512	0.273–0.952	

Nodes status and RANK expression are independent prognostic indicators for early bone metastasis development (*P* = 0.029 and 0.037, respectively).

C.I., Confidential Interval; RR: relative risk.

## Discussion

RANK / RANK-L is a largely studied pathway (mainly in the preclinical setting) and that its key role in the metastatic process to bone was hypothesized and investigated before by using in vivo models (16,21,22). In the clinical setting, several groups found an association between high expression of RANK/RANKL/OPG in the primary tumor (e.g. prostate, HCC, not in breast cancer) and propensity to develop bone metastasis (18,20). Our group recently demonstrated that RANK is expressed by human solid tumors (mostly breast, colorectal, renal, lung, and prostate cancer) with high concordance between bone metastasis and corresponding primary tumor (23). These data highligh the central role of RANK/RANKL/OPG pathway as therapeutic target in bone metastasis management. The discovery of the role of RANK/RANKL/OPG pathway in bone reabsorption produced great expectations. A therapeutic targeting could help in preventing skeletal related events in breast cancer patients with bone metastases, preserving bone health in patients receiving cancer treatments that induce bone loss (CTIBL) and reducing the risk of disease recurrence in adjuvant setting. Herein, we report our findings of the prognostic role of RANK/RANKL/OPG in a microarray dataset of 295 early-stage breast cancer patients. In particular, high RANK and low OPG levels in primary tumors are predictive of worst prognosis. At the same time, the expression of both genes examined was correlated with the main clinicopathological and molecular features of primary breast tumors. A higher RANK gene expression has been found in the “poor prognosis”, >2 cm, estrogen receptor negative and G3 tumors. On the other hand, a higher OPG expression was found in the “good prognosis” signature population and G1 tumors, according to Poznak et al [24]. Positive correlation between OPG and ER IHC expression in breast tumors has been reported by Van Poznak et and Cross et al [17], [24]. Conversely, RANKL appeared to be inversely correlated with ER in IHC analysis [24]. Our microarray results do not show a significant correlation between ER status and OPG/RANKL expression. However, a comparison between results from two different methodic (IHC and microarray) has intrinsic limitations. Unfortunately, we do not have IHC data for OPG and RANKL in our series. Further studies will be needed. There is no correlation between age and expression of RANK, RANKL or OPG in NKI dataset. Unfortunately, we do not possess clinical data about the menopausal status of these patients.

Interestingly, a recent model has been proposed in which surges of progesterone occurring during the reproductive cycle and pregnancy prompt mammary stem cells (MaSCs) proliferation, providing a window during which MaSCs (ER−/PR−) are targets for oncogenic mutations [25]. This paracrine effector of progesterone could be RANKL from luminal cells binding to its receptor on MaSCs.

In fact, Joshi et al. showed that both luminal and basal cells surrounding the MaSCs niche significantly increased PR expression following treatment with 17β-oestradiol and progesterone [26]. They also observed profound upregulation of Wnt4 and RANKL in luminal cells as well as induction of their respective receptors Lrp5 and Rank in the MaSC-enriched basal cell population. To note, both RANK and WNT4a pathways are known mediators of progesterone driven paracrine effects in the mammary gland. Moreover, Asselin-Labat et al. found, during mid-pregnancy, a marked increase in RANKL expression in the luminal cell population, and the RANK receptor and the RANK signaling target Id2 in the MaSC-enriched basal cell subset [27]. Treatment with a RANKL inhibitor suppressed the clonogenic activity of this population.

Moreover, Beleut et al. found that RANKL elicits proliferation in the mammary gland by a paracrine mechanism [28]. Ablation of RANKL in the mammary epithelium blocks progesterone-induced morphogenesis, and ectopic expression of RANKL in mammary epithelial cells (MECs) completely rescues the PR−/− phenotype. Systemic administration of RANKL triggers proliferation in the absence of PR signaling, and injection of a RANK signaling inhibitor interferes with progesterone induced proliferation.

These findings open the scenario for a major role of RANK/RANKL pathways in the mammary gland compartments. All these data support the emerging prognostic role of this pathway in breast cancer patients.

We focused the experimental phase of this study on RANK, because of its strong correlation with clinical prognostic factors and outcome found in the microarray analysis. NKI dataset's clinical annotations were lacking of the SDFS data, which we followed in our dataset of 93 patients. We observed that higher IHC RANK expression in primary tumors is significantly correlated with higher risk to develop bone metastases and with a shorter SDFS.

These results are consistent with the recent preclinical investigations in both *in vitro* and *in vivo* murine models mimicking bone metastatization. In fact, based on the high constitutive RANK expression in breast cancer specimens and cell lines, recent data suggest that RANK expression status of cancer cells determines whether tumors predominantly migrate into bone, whereas the corresponding ligand RANKL is abundantly expressed. The correlation of high RANK expression with osteotropism in murine models was demonstrated across diverse tumor cell types, including breast cancer and melanoma [29]. Blocking RANKL-RANK signaling in these mice by OPG administration led to a reduction of the skeletal tumor burden by 50% and prevented tumor-induced paralysis [29]. For these reasons, according to our original discoveries, we can support a potential important functional role of RANK expression on primary breast cancer in the complex bone metastatization process.

In order to identify potential molecular mechanisms involved in determining the site of relapse, Smid et al. [30] mapped differentially expressed genes from 107 primary breast tumors from lymph node negative patients at the time of diagnosis which experienced relapse. A panel of 69 genes was identified as significantly differentially expressed between patients who relapsed to bone versus those who relapsed elsewhere. The most differentially expressed gene was TFF1. Another approach was used by Kang et al. in order to find mediators of breast cancer metastasis to bone by using MDA-MB-231 cells [31]. Over-expression of the bone metastasis specific gene set (named genes encoding C-X-C chemokine receptor [CXCR]4, IL-11, connective tissue growth factor, and matrix metalloproteinase [MMP]-1), along with the osteopontin gene and five genes members of FGF receptor pathway (FGF5, SOS1, DUSP1, FGFR3, and DUSP4) in various combinations, considerably enhanced the metastatic potential of breast cancer cells to bone. Anyway, each of these genes, when expressed individually, failed to confer high skeletal tropism. However, many of the identified classifiers and gene targets in the various studies are largely non-overlapping, raising questions about their biologic significance and clinical implications. Other studies have reported similar gene expression-based approaches [21], [32]. Thanks to our two-steps approach, we observe that RANK expression has a prognostic value in a microarray dataset of a breast cancer population, even though the lack of availability of SDFS in that dataset was a limit. However, our IHC results are the first demonstration of the role of RANK expression in primary tumors as a predictive marker of bone metastasis occurrence and SDFS in a large population of breast cancer patients. We think that our findings can be considered a trigger for perspective larger studies in this setting.

### Conclusions

Many studies strongly examined and confirmed the link between RANK/RANKL/OPG pathway and propensity to metastasize to the bone. Investigating the RANK/RANKL/OPG pathway might open new scenarios in predicting bone disease recurrence and prognosis and potentially preventing bone metastatization in RANK-expressing early breast cancer patients. The availability of a monoclonal antibody toward RANKL (denosumab) strengthens the clinical relevance of this work. Denosumab is a breakthrough fully human monoclonal antibody now approved by both the FDA and EMA. It had been fast tracked by FDA for treatment and prevention of postmenopausal osteoporosis, and treatment and prevention of bone loss in hormone treated prostate and breast cancer patients [33], [34]. The modulation of the gene and protein expression trough anti-RANKL therapy at primary tumor level might modify the physiopathology of bone metastases. In this regard, our work represents a strong biological rational supporting the ongoing randomized, double-blind, placebo-controlled, multi-center phase 3 study evaluating Denosumab as adjuvant treatment for women with early-stage breast cancer at high risk of recurrence. In the next future Denosumab could be tested to verify the role of RANKL/RANK pathway in preventing skeletal migration and metastases in the subpopulation of RANK expressing early breast cancer patients.

## Materials and Methods

### Microarray analysis

We used one publicly available microarray dataset of 295 patients with primary breast cancer from NKI cohort [35]. Raw data and complete clinical data were downloaded from the Rosetta Web site (http://www.rii.com). Clinico-molecular characteristics of the patients are shown in Table 1.

Raw data have been imported in Partek Genomic Suite 6.4 software (Partek Inc., St. Louis, MO), transformed in log2 and normalized by quantile normalization. Principal Component Analysis (PCA) was used to verify the quality of data. The log2 transformed data for the following probe sets have been evaluated: RANK, RANKL, OPG. Population has been stratified on basis of the main available prognostic factors for breast cancer: Estrogen Receptor (ER) status, tumor size and histological grading. Moreover, the 70-gene prognostic profile (van't Veer signature) has been used to divide the population in “Good prognosis” and “Poor prognosis” group. The classification in molecular subtypes by Sorlie et al. has been also considered [36].

### Patients of the experimental phase

We investigated RANK expression in primary tumors by immunohistochemistry (IHC) and its possible association with clinicopathological parameters, bone recurrence and survival. The study retrospectively enrolled women with confirmed histological diagnosis of breast cancer who underwent radical surgery. In particular, surgical biopsy samples of 93 patients with intraductal carcinoma (IDC) or intralobular carcinoma (ILC) were retrieved from the surgical archives of the histopathology departments of Campus Bio-Medico University (Roma), Institute for Cancer Research and Treatment (Torino), “Ospedale S.Giovanni di Dio” (Napoli), “Policlinico S.Matteo” (Pavia), “Ospedale di Galliera” (Genova) and “Ospedale Fatebenefratelli” (Milano). The study was approved by the Institutional Review Boards of the above mentioned institutions/hospitals. No informed consent statement from patients was obtained, since samples collected at the moment of the diagnosis were retrospectively retrieved, made anonymous and coded. The ethics committees approved this procedure.

Key inclusion criteria were the availability of a follow-up at least of 24 months from the surgery and absence of metastatic disease at the diagnosis of primary tumors. Exclusion criteria were neoadjuvant therapy and not sufficient tissue available for IHC. All patient records were collected into a common database. A complete patient record contained information on clinicopathological features of the tumor site of recurrences, SDFS and OS. Follow-up data were available for all the cases, with a median follow-up of 43 months. Clinicopathological information were retrieved from the pathology and medical records; patients were stratified into four groups: 1) patients who developed bone lesions as first metastatic site; 2) patients who developed bone lesions after the appearance of visceral metastasis, 3) and 4) patients who did not develop bone metastasis (only visceral metastasis and without any metastases, respectively). Detailed clinical data of these patients are provided in Table 2.

### Immunohistochemical analysis

Representative tumor blocks were sectioned at 3 µm thickness. IHC was performed by the streptavidin-biotin method. Endogenous peroxidase in the section was blocked by incubating them in 3% hydrogen peroxide. A mouse monoclonal antibody against RANK protein (clone 80707, R&D Systems, Inc.) was used at concentration of 25 µg/mL. This antibody has been used and validated previously by our group and others [23], [37]. Sections were incubated with LSAB2 (Dakocytomation). 3-3′-diaminobenzidine (DAB) was used for color development and haematoxylin was used for counterstaining. Scoring for RANK was based on relative intensities of staining of tumor cells with reference to the normally present RANK staining of tissue associated macrophages. These internal references were used as internal positive controls between slides and samples as well as for the staining procedure. Notably, we performed additional immunofluorescence experiments on formalin fixed and paraffin embedded tissue sections to further validate our internal positive control, in particular the ability of the anti-RANK antibody to bind to macrophages (Figures S1 and S2).

Cancer lesions tissue was then evaluated by comparison with the internal controls. Staining intensity was graded as absent (0), positive but less (+1), like (2+) or more (3+) intense than internal control tissue. Samples with regions of heterogeneous staining intensities were scored and the percentage of staining intensity for each area was recorded. Overexpression of RANK protein was considered when neoplastic cells showed 2+ and 3+ immunostaining intensity. The average number of tumor cells overexpressing RANK was ∼50% (48,3%), therefore 50% was chosen as cut-off for binomial classification of tumor samples. An immunostaining intensity of 2+ and 3+ in more than 50% of cancer cells was considered as the cut-off point to consider a sample as “positive”. Immunostaining was assessed by two independent pathologists blinded to clinical characteristics and outcomes. Agreement in immunohistochemical evaluation between the two observers was >90% (Kappa value: 0.961).

### Statistical analysis

Log2 normalized expression data for RANK/RANKL/OPG probesets have been compared in the different groups by using the unpaired *t* test and one-way ANOVA test (for two unpaired groups or more than two groups, respectively). 

Descriptive analyses were performed using the median values and the corresponding 95% Confidence Interval (CI). The association between variables in the 93 patients dataset was performed using the chi square test.

In order to analyze the correlation between RANK/RANKL/OPG pathway and outcome in 295 breast cancer patients we divided the population in three equally numerous groups depending on the expression level of each probesets (high, medium or low), according to previously used methods [38].

For the survival analysis, tumors with high (H) and medium (M) RANK mRNA level were grouped together and are indicated as possessing high RANK (H) vs low RANK (L). OPG expression was dichotomized as low (L+M) vs high (H).

DFS and SDFS analysis were calculated as the period from the date of diagnosis to the first observation of any metastasis and bone metastases, respectively. The OS time was calculated as the period from the date of diagnosis until death from any cause or last follow-up. Univariate analysis of OS, DFS and SDFS has been estimated according to the Kaplan-Meier method and analyzed by Log-rank (Mantel-Cox) Test [39]. Hazard ratios and 95% confidence intervals (CIs) were estimated by use of a stratified Cox regression analysis.

The Cox proportional hazards model was applied to the multivariate survival analysis [40].

Statistical analysis has been performed with GraphPad Prism software version 5.00 (GraphPad Software Inc., San Diego, CA, USA) and SPSS software (version 17.00, SPSS, Inc.). A two-sided *P*-value<0.05 has been considered statistically significant.

## Supporting Information

Figure S1 **Immunofluorescence staining with anti-RANK and anti-CD68 antibodies of a breast cancer section.** A RANK positive breast cancer section was stained with anti-RANK (B, green) and anti-CD68 (C, red) antibodies. The merged image (D) shows that the CD68 positive cell is also positive for RANK, confirming RANK expression by macrophages. (A) = DAPI staining.
(TIF)

Figure S2 **Immunofluorescence staining with anti-CD68 and anti-AE1-AE3 cytokeratin antibodies of a breast cancer section.** Immunofluorescence staining for CD68 (red) and AE1-AE3 cytokeratin (green) antibodies shows staining that macrophages are completely negative to AE1-AE3 antibody.
(TIF)
