# Supplementary figures and images for: Receptor Activator of NF-kB (RANK) Expression in Primary Tumors Associates with Bone Metastasis Occurrence in Breast Cancer Patients
Source: PLoS One. 2011 Apr 29;6(4):e19234. doi: 10.1371/journal.pone.0019234 (PMC3084800; doi:10.1371/journal.pone.0019234)

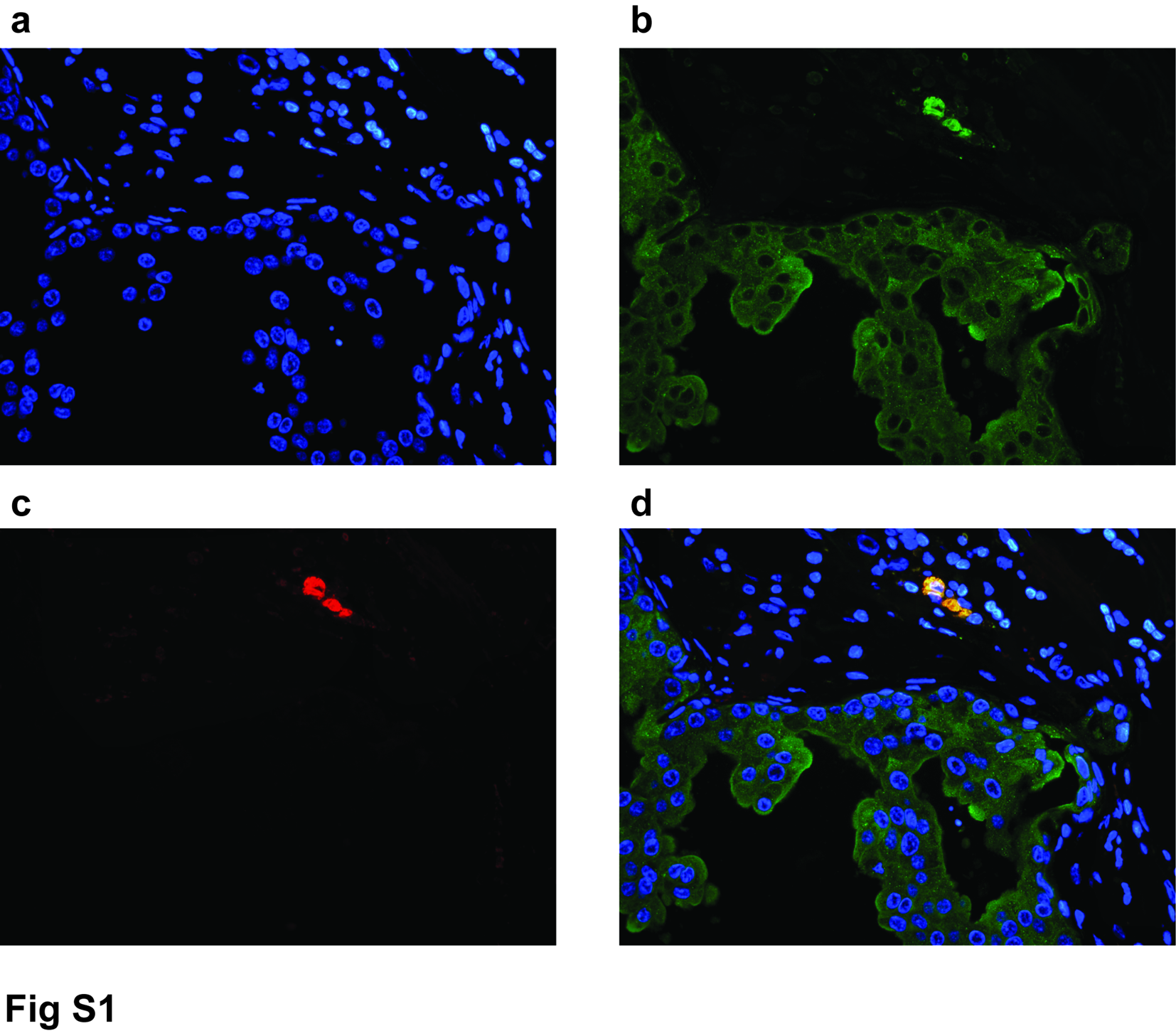

Supplement: Figure S1 — Immunofluorescence staining with anti-RANK and anti-CD68 antibodies of a breast cancer section. A RANK positive breast cancer section was stained with anti-RANK (B, green) and anti-CD68 (C, red) antibodies. The merged image (D) shows that the CD68 positive cell is also positive for RANK, confirming RANK expression by macrophages. (A) = DAPI staining. (TIF) [file pone.0019234.s001.tif]

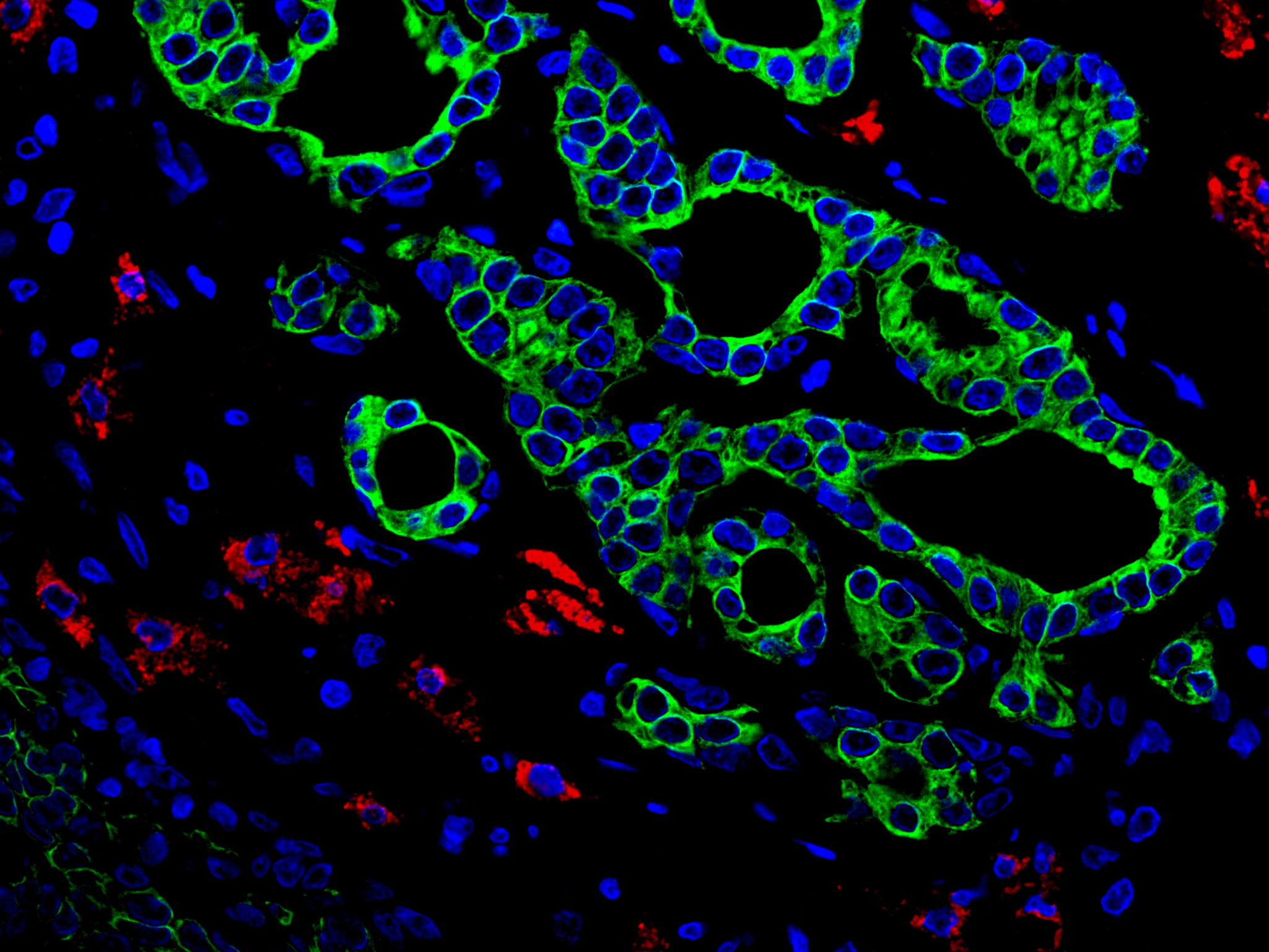

Supplement: Figure S2 — Immunofluorescence staining with anti-CD68 and anti-AE1-AE3 cytokeratin antibodies of a breast cancer section. Immunofluorescence staining for CD68 (red) and AE1-AE3 cytokeratin (green) antibodies shows staining that macrophages are completely negative to AE1-AE3 antibody. (TIF) [file pone.0019234.s002.tif]
